# Apoptotic Killing of HIV-1–Infected Macrophages Is Subverted by the Viral Envelope Glycoprotein

**DOI:** 10.1371/journal.ppat.0030134

**Published:** 2007-09-28

**Authors:** Simon Swingler, Angela M Mann, Jin Zhou, Catherine Swingler, Mario Stevenson

**Affiliations:** Program in Molecular Medicine, University of Massachusetts Medical School, Worcester, Massachusetts, United States of America; National Institute of Allergy and Infectious Diseases, United States of America

## Abstract

Viruses have evolved strategies to protect infected cells from apoptotic clearance. We present evidence that HIV-1 possesses a mechanism to protect infected macrophages from the apoptotic effects of the death ligand TRAIL (tumor necrosis factor–related apoptosis-inducing ligand). In HIV-1–infected macrophages, the viral envelope protein induced macrophage colony-stimulating factor (M-CSF). This pro-survival cytokine downregulated the TRAIL receptor TRAIL-R1/DR4 and upregulated the anti-apoptotic genes Bfl-1 and Mcl-1. Inhibition of M-CSF activity or silencing of Bfl-1 and Mcl-1 rendered infected macrophages highly susceptible to TRAIL. The anti-cancer agent Imatinib inhibited M-CSF receptor activation and restored the apoptotic sensitivity of HIV-1–infected macrophages, suggesting a novel strategy to curtail viral persistence in the macrophage reservoir.

## Author Summary

Much of our understanding regarding mechanisms of HIV-1 persistence has been derived from studies with lymphocytes. However, mechanisms governing persistent infection of macrophages are less well understood. We investigated whether HIV-1 modulates macrophage function in a way that promotes their persist infection. We focused on a cytokine called macrophage colony-stimulating factor (M-CSF), because this pro-survival factor is induced upon infection by HIV-1. We found that the viral envelope gene was necessary for M-CSF induction of macrophages. M-CSF upregulated anti-apoptotic genes in macrophages and restricted the expression of the death receptor (tumor necrosis factor–related apoptosis-inducing ligand [TRAIL]-R1). As a consequence, HIV-1–infected macrophages were resistant to the apoptotic effects of TRAIL. If M-CSF was blocked by antibody or if the anti-apoptotic genes were silenced by RNA interference, the apoptotic sensitivity of HIV-1–infected macrophages was restored. Also, the anti-cancer drug Imatinib, which impairs activation of the M-CSF receptor, promoted the death of HIV-1–infected macrophages but not of uninfected macrophages. We believe that HIV-1 regulates M-CSF to extend macrophage survival and promote viral persistence in the host. Agents that interfere with M-CSF signaling, such as Imatinib, warrant further examination for activity against macrophage reservoirs in vivo*.*

## Introduction

The envelope glycoproteins of primate lentiviruses harbor domains that mediate the interaction with receptor/co-receptor proteins on the surface of susceptible cells and promote fusion between viral and cellular membranes during virus entry [1]. However, some studies suggest that activities of human lentiviral envelope proteins extend beyond their role in viral entry. For example, the envelope protein signals through receptor and co-receptor molecules after envelope binding, an activity that may alter target cell function to increase the cell's permissivity to virus infection [2,3]. In addition, the HIV-1 envelope, like other retroviral envelope proteins [4], contains domains that interact with the intracellular signal transduction machinery to promote changes in cell function [5,6], such as the induction of pro-inflammatory cytokines [7].

The prevention of apoptosis of the infected cell is an important modification to host cell function, particularly during chronic viral infections [8]. As a consequence, viruses have evolved measures that either confer resistance to apoptotic cell death or kill the cells delivering the apoptotic signal. Adenoviruses encode RID proteins that promote the internalization of death receptors for Fas, tumor necrosis factor–related apoptosis-inducing ligand (TRAIL), and tumor necrosis factor receptor 1 (TNFR1), thereby allowing infected cells to withstand these apoptotic stimuli [9–11]. As part of a counterattack strategy, human cytomegalovirus upregulates death-inducing ligands on the infected cell, triggering apoptosis of human cytomegalovirus–specific T cells [12].

Most of the data on HIV-1 immune evasion strategies have been derived from experiments in lymphocytes. For example, the HIV-1 Nef gene has been shown to block the function of ASK 1 (apoptosis signal-regulating kinase 1) in infected lymphocytes to protect these cells from Fas and TNFR-mediated apoptosis [13]. At the same time, HIV-1 Nef induces Fas ligand expression on infected cells to kill cytotoxic T cells expressing Fas on the cell surface [14]. Despite the existence of viral mechanisms to protect lymphocytes from the host apoptotic response, productively infected lymphocytes are rapidly cleared by the cytopathic effects of virus infection [15]. By comparison, the turnover of infected macrophages is slow. Extrapolation of the decay characteristics of plasma virions during highly active antiretroviral therapy suggests that the half-life of the infected macrophage reservoir in the tissues is on the order of 2–4 wk [15]. However, a greater half-life is suggested by studies with highly pathogenic SHIV (simian immunodeficiency virus [SIV]/HIV chimera) variants, which demonstrated no decay in the macrophage reservoir over a 3–4-mo interval when viral spread was prevented by the antiretroviral PMPA [16]. Whether HIV-1 has a mechanism to sustain the persistence of infected macrophages is unknown.

## Results

### The HIV-1 Envelope Regulates the Pro-Survival Cytokine M-CSF

**Figure 1:**
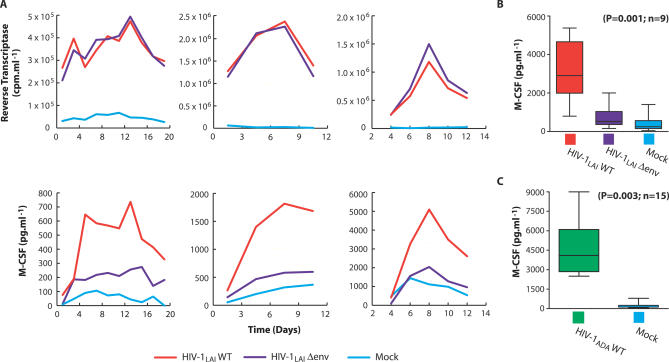
HIV-1 Envelope-Dependent M-CSF Induction by Infected Macrophages (A) Levels of virus (upper panels) and M-CSF (lower panels) production by macrophages following infection with VSV-envelope pseudotyped X4 HIV-1 variants (HIV-1_LAI_) containing intact (WT) or defective (Δenv) HIV-1 envelope genes. Results obtained with macrophages from three independent donors are shown. (B) Statistical analysis of peak mean M-CSF release by pseudotyped X4-tropic HIV-1_LAI_WT, HIV-1_LAI_Δenv or mock-infected macrophages (ANOVA; error bars, SEM), and (C) by macrophages infected with R5-tropic HIV-1_ADA_WT or mock-infected (*t*-test; error bars, SEM).

HIV-1–infected macrophages release the pro-survival cytokine M-CSF (macrophage colony-stimulating factor) and the β-chemokines MIP-1α and MIP-1β [17,18]. To characterize the biological role of M-CSF in HIV-1 replication, we initially identified the viral gene product responsible for its induction. To restrict viral replication to a single cycle, macrophages were infected with vesicular stomatitis virus (VSV)–pseudotyped, X4-tropic viruses containing inactivating mutations in structural and accessory genes. Levels of virus production (extracellular reverse transcriptase [RT] activity) and M-CSF release were monitored at different intervals after infection. While the accessory proteins Vpu, Vif, Nef, and Vpr were relatively dispensable for the induction of M-CSF (unpublished data), the release of M-CSF by macrophages infected with an HIV-1 envelope-minus variant (HIV-1_LAI_Δenv) was impaired relative to macrophages infected with a wild-type virus, despite indistinguishable levels of virus production in wild-type– and Δenv-infected cultures (Figure 1A). There was a statistically significant (*p* = 0.001) relationship between envelope and M-CSF when peak M-CSF levels in HIV-1_WT_, HIV-1Δ env, and mock-infected macrophages from nine independent experiments were compared (Figure 1B). In addition, during infection of macrophages with wild-type R5-tropic HIV-1, M-CSF production was significantly induced over mock-infected cultures (*p* = 0.003, *n* = 15; Figure 1C).

The HIV-1 envelope protein induces signals through the chemokine receptors that influence the expression of cellular genes [19,20]. Macrophage infection by HIV-1 is mediated primarily by the co-receptor CCR5 [1]. Since M-CSF was induced by X4-tropic viruses that are unable to use CCR5 on macrophages (Figure 1A and 1B), the induction appeared to be independent of co-receptor use. Macrophages infected with a pseudotyped HIV-1 variant that harbored a mutation in the CD4 binding epitope of envelope [21] also induced M-CSF production. However, M-CSF was not induced when macrophages were pulsed with gradient-purified R5-tropic HIV-1_ADA_ virions or with HIV-1_ADA_ Δenv virions (Figure S1). Collectively, these results indicate that the induction of M-CSF by the viral envelope glycoprotein was independent of receptor or co-receptor interactions. In addition, de novo synthesized envelope, but not virion-associated envelope, induced M-CSF production.

### HIV-1 Envelope Restricts Expression of Death Receptors on Infected Macrophages

**Figure 2:**
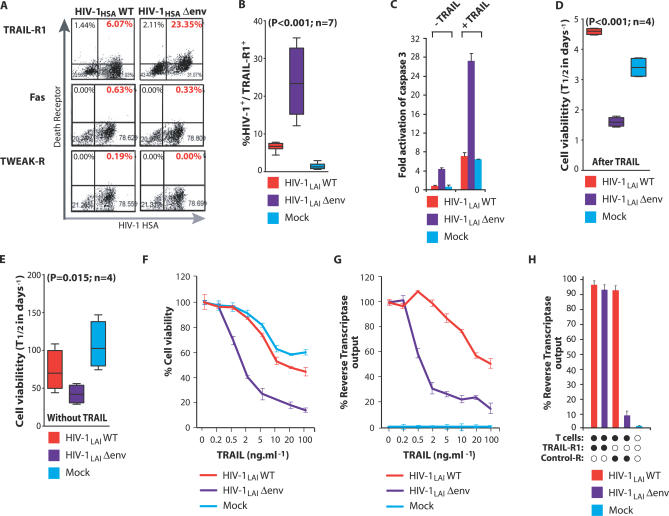
Death Receptor Expression on Macrophages Infected with Wild-Type or Envelope-Minus HIV-1 Variants (A) Macrophages were infected with VSV-pseudotyped wild-type HIV-1 (HIV-1_HSA_WT) or envelope-minus (HIV-1_HSA_Δenv) variants expressing HSA in place of Vpr. Levels of TRAIL-R1, Fas, and TWEAK-R expression on infected (HSA positive) cells was determined by flow cytometry. (B) Statistical analysis of mean TRAIL-R1 expression from macrophages from seven donors infected with pseudotyped HIV-1_HSA_ wild-type or envelope-minus viruses relative to mock-infected (ANOVA; error bars, SEM). (C) The HIV-1 envelope negates macrophage susceptibility to apoptosis by TRAIL. Macrophages were infected with pseudotyped wild-type or Δenv minus HIV-1_LAI_ variants. At 8 d post-infection, cultures were incubated with soluble TRAIL (100 ng/ml^−1^). Apoptosis was determined by ELISA for active (cleaved) caspase 3. The viral envelope increases macrophage survival in the presence of TRAIL. Eight days after infection with pseudotyped HIV-1_LAI_ wild-type or Δenv viruses, macrophages were maintained with (D) or without (E) soluble TRAIL (100 ng/ml^−1^), and the percentage of viable cells (cell death ELISA) remaining over time was determined. Macrophage half-life was calculated from the linear regression slope over 24 h, compared by ANOVA, and expressed as mean value in days ± SEM. Concentrations of TRAIL required to impact viability and virus output in macrophages infected with wild-type and Δenv HIV-1 variants. Macrophages infected with HIV-1_LAI_ wild-type or Δenv viruses were incubated with increasing concentrations of soluble TRAIL and cell viability (F) and virus production (G) were determined after 16 h. (H) Activated CD8^+^ T cells selectively suppress virus production by HIV-1 Δenv-infected macrophages. Wild-type and Δenv HIV-1–infected macrophages were incubated with anti-CD3/CD28 stimulated autologous CD8^+^ TRAIL^+^ T lymphocytes for 4 h. Virus production was determined after an additional 24 h. T cells were pre-incubated with recombinant TRAIL-R1 or control receptor (5 μg/ml^−1^) for 1 h prior to co-culture.

Since cytokines can counteract apoptotic signals [22,23], we analyzed whether the differential expression of M-CSF in HIV-1 wild-type– and Δenv-infected macrophages affected the expression of apoptotic mediators in these cells. When analyzed using pathway-specific gene arrays, macrophages infected with a Δenv virus but not with a wild-type virus expressed greatly elevated mRNA levels for the TRAIL Death Receptor TRAIL-R1/DR4 (Figure S2). This reflected an increase in surface TRAIL-R1 on HIV-1 Δenv-infected macrophages compared with wild-type–infected macrophages (23% TRAIL-R1 positive for Δenv versus 6% for wild-type–infected macrophages; Figure 2A). Furthermore, in a comparison of seven independent experiments, a statistically significant difference in TRAIL-R1 expression was observed following infection of macrophages with envelope minus virus relative to wild-type– and mock-infected macrophages (*p* < 0.001; Figure 2B). However, expression of death receptors for Fas ligand and TWEAK (tumor necrosis factor–like weak inducer of apoptosis) were not affected by HIV-1 infection (Figure 2A).

We next examined whether the differential expression of TRAIL-R1 on HIV-1 wild-type– and Δenv-infected macrophages conferred differential sensitivity to TRAIL. Macrophages infected with a Δenv virus were highly susceptible to TRAIL killing, as evidenced by activation of caspase 3, a hallmark of TRAIL-dependent apoptosis (Figure 2C). In contrast, caspase 3 levels in wild-type–infected macrophages in both the presence and absence of TRAIL were similar to levels in uninfected macrophages (Figure 2C). TRAIL also selectively curtailed the survival of macrophages infected with a Δenv virus. In the presence of TRAIL, the half-life of macrophages infected with a Δenv virus was ∼1.7 d, compared with ∼4.6 d for wild-type virus and ∼3.5 d for mock-infected cells (Figure 2D). In the absence of TRAIL, Δenv-infected macrophages also possessed the shortest half-life, ∼38 d, while wild-type– or mock-infected macrophages exhibited a half-life of ∼70 d and ∼100 d, respectively (Figure 2E). We also determined the relative concentrations of TRAIL required to impact viability and virus output of macrophage cultures infected with wild-type and Δenv viruses. In macrophages infected with a Δenv HIV-1 variant, a TRAIL concentration of 3 ng/ml^−1^ produced a 50% reduction in cell viability and virus output (Figure 2F and 2G). In contrast, more than 30 ng/ml^−1^ and 50 ng/ml^−1^ of TRAIL was required for a 50% reduction in viability and virus output, respectively, from wild-type–infected macrophages (Figure 2F and 2G). HIV-1 envelope also rendered infected macrophages resistant to TRAIL on the surface of activated T lymphocytes. When activated CD8^+^ T cells (∼16% TRAIL positive; unpublished data) were co-cultured with infected macrophages, virus output from HIV-1 Δenv-infected macrophages was reduced by ∼90%, but virus output from wild-type–infected macrophages was not affected (Figure 2H). Collectively, these results indicate that HIV-1–infected macrophages are resistant to TRAIL and that the viral envelope glycoprotein is required for this resistance.

### HIV-1 Envelope Regulates TRAIL Sensitivity via M-CSF

**Figure 3:**
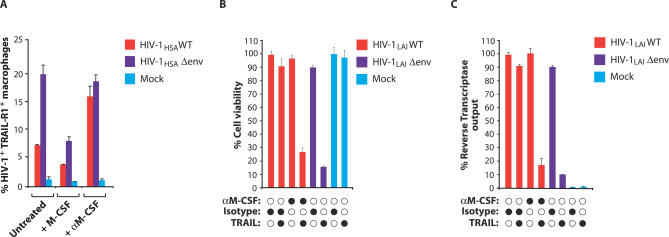
M-CSF Modulates TRAIL-R1 Expression (A) HIV-1 wild-type–, Δenv-, or mock-infected macrophages were analyzed by flow cytometry 16 h after treatment with recombinant M-CSF (5,000 pg/ml^−1^) or with a neutralizing antibody to M-CSF (error bars, SD). (B,C) HIV-1 envelope is required for the resistance of infected macrophages to TRAIL. HIV-1 wild-type– and Δenv-infected macrophages were incubated with neutralizing antibody to M-CSF for 16 h. Cultures were then treated with soluble TRAIL (100 ng/ml^−1^). Cell viability (B) and virus output (C) were determined after 24 h by MTT assay and RT production, respectively.

The viral envelope glycoprotein induced the production of M-CSF in infected macrophages (Figure 1). Since cytokines can promote cell survival by opposing apoptotic signals [22,23], we examined whether M-CSF was required for the resistance of wild type–infected macrophages to TRAIL. When M-CSF was neutralized in macrophage cultures infected with wild-type HIV-1, surface TRAIL-R1 expression increased (Figure 3A). In parallel, neutralization of M-CSF in macrophage cultures infected with wild-type R5-tropic HIV-1 elevated TRAIL-R1 expression (Figure S4A–S4C). Conversely, addition of M-CSF to the culture downregulated TRAIL-R1 expression on macrophages infected with an envelope defective virus (Figure 3A). M-CSF neutralization restored TRAIL sensitivity to macrophages infected with wild-type virus; macrophage viability was reduced by 75% and virus output by 85% when M-CSF was neutralized in these cultures (Figure 3B and 3C). M-CSF neutralization rendered wild-type virus–infected macrophages as sensitive to TRAIL as macrophages infected with Δenv virus. Together, these results demonstrate that M-CSF is required for the ability of the viral envelope glycoprotein to confer TRAIL resistance to infected macrophages.

### The Anti-Apoptotic Genes Bfl-1 and Mcl-1 Mediate TRAIL Resistance

**Figure 4:**
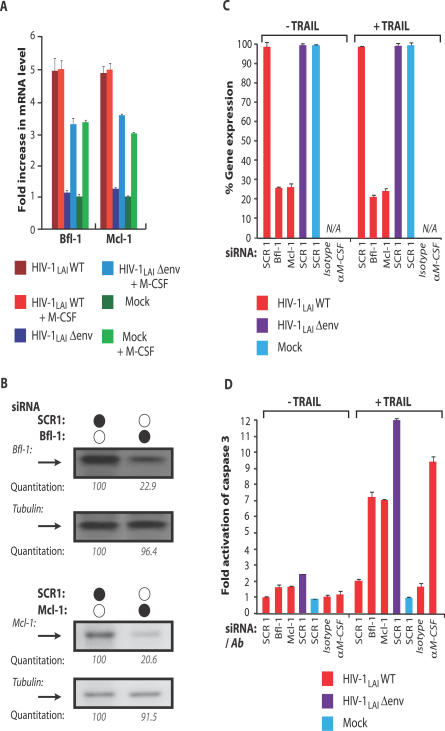
M-CSF Upregulates Host Anti-Apoptotic Genes to Mediate Resistance of Infected Macrophages to TRAIL (A) mRNA levels for the anti-apoptotic genes Bfl-1 and Mcl-1 were measured by ribonuclease protection assay in HIV-1 wild type– and Δenv-infected macrophage cultures incubated in the presence or absence of M-CSF. (B) RNAi effectively mediated substantial reduction in Bfl-1 and Mcl-1 protein levels in human macrophages. Protein levels were determined by Western blotting and normalized to signals from tubulin. (C) RNAi-mediated Bf1–1 and Mcl-1 knockdown of mRNAs. mRNA levels, determined by quantitative RT-PCR, are shown as percentage relative to a scrambled siRNA (SCR1) (error bars, SD). (D) Bfl-1 and Mcl-1 are required for protection from TRAIL-mediated apoptosis. Macrophages were transfected with Mcl-1, Bfl-1, or a nontargeting control (SCR1) siRNA. Sixteen hours later, macrophages were treated with soluble TRAIL, or an antibody to M-CSF, and apoptosis was determined by ELISA for active (cleaved) caspase 3 (error bars, SD).

We next set out to identify host factors through which the viral envelope regulated the susceptibility of infected macrophages to TRAIL. We targeted the mRNA analysis to cellular genes involved in the TRAIL apoptosis pathway. We predicted that genes that mediate the protective effect of the viral envelope would also be upregulated by M-CSF. Two genes, Bfl-1 and Mcl-1, which inhibit mitochondrial-dependent apoptosis [24, 25], were upregulated in macrophages infected with wild-type HIV-1 and in mock-infected macrophages that had been stimulated with M-CSF (Figure 4A). However, Bfl-1 and Mcl-1 were not upregulated in macrophages infected with a Δenv virus (Figure 4A). To determine whether these anti-apoptotic genes were necessary for HIV-1 to render macrophages resistant to TRAIL, the resistance of infected macrophages to TRAIL was examined after silencing these genes by RNA interference. In macrophages, RNA interference achieved a 70%–75% knockdown of Bfl-1 and Mcl-1 proteins and mRNA when assessed by Western blotting and quantitative RT-PCR, (reverse transcription PCR), respectively (Figure 4B and 4C). Silencing of either Bfl-1 or Mcl-1 restored the susceptibility of wild-type–infected macrophages to TRAIL (Figure 4D). Levels of caspase activation in wild-type–infected macrophages in which Bfl-1 or Mcl-1 had been silenced approached levels observed in wild-type–infected macrophages in which M-CSF had been neutralized (Figure 4D). In the absence of TRAIL, neither Bfl-1/Mcl-1 silencing nor M-CSF neutralization had a significant impact on the levels of macrophage apoptosis (Figure 4D). Three other host genes, cIAP-1, cIAP-2, and XIAP, which antagonize caspase activation [24, 26], were upregulated by the viral envelope during the first days of macrophage infection, prior to the induction of M-CSF release. From similar RNA interference experiments, these IAP genes conferred moderate resistance of wild-type–infected macrophages to TRAIL-mediated apoptosis (Figure S3 and unpublished data). Collectively, these results demonstrate that HIV-1 envelope glycoprotein in macrophages induces M-CSF, which in turn opposes the apoptotic effects of TRAIL by inducing the anti-apoptotic proteins Bfl-1 and Mcl-1.

### Imatinib Blocks M-CSF Function and Restores TRAIL Sensitivity to Infected Macrophages

**Figure 5:**
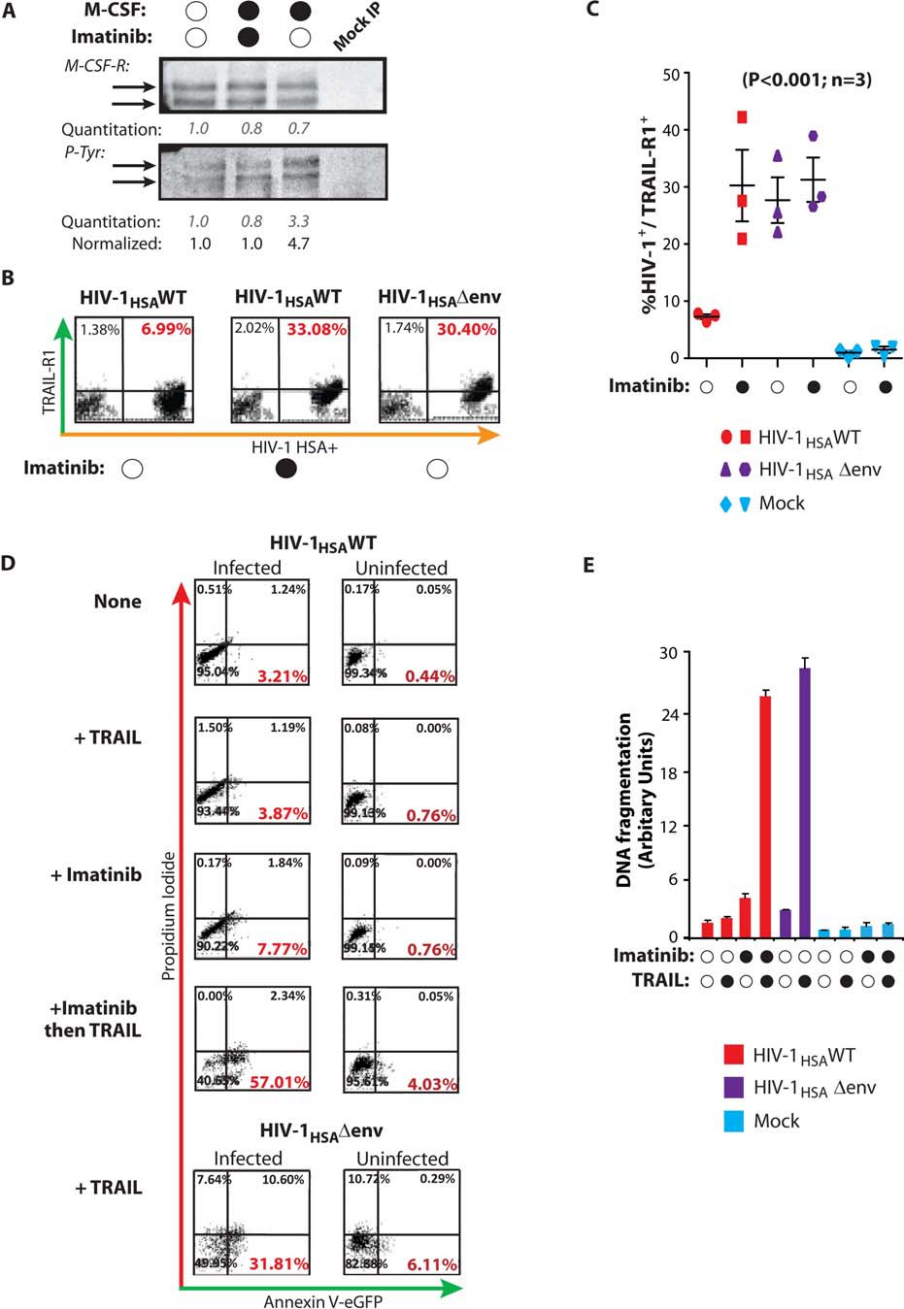
Imatinib Blocks M-CSF Signaling and Restores TRAIL Sensitivity of Infected Macrophages (A) Imatinib inhibits M-CSF–dependent autophosphorylation of the M-CSF receptor. Macrophages were incubated for 16 h with 2 μM Imatinib mesylate before stimulation with recombinant M-CSF for 5 min. The M-CSF receptor was immunoprecipitated from cell lysates, and M-CSF–dependent tyrosine autophosphorylation was determined by Western blotting and densitometry. (B) Imatinib upregulates TRAIL-R1 expression on wild-type–infected macrophages. HIV-1 wild-type– and Δenv-infected macrophages were incubated with Imatinib for 16 h and TRAIL-R1 levels were determined by flow cytometry. (C) Macrophages from three donors were treated as in (B), and statistical analysis was performed on the mean expression of TRAIL-R1 in response to Imatinib (ANOVA; error bars, SEM). (D) Imatinib restores sensitivity to TRAIL. Macrophages were incubated with Imatinib for 16 h, challenged with soluble TRAIL, or both. Apoptotic HIV-1–infected and uninfected cells in the same cultures were determined 6 h later by Annexin V and propidium iodide staining and flow cytometry. Apoptotic cells were Annexin V^+^, propidium iodide^−^ (lower right quadrant). Imatinib renders HIV-1 wild-type–infected macrophages susceptible to TRAIL-mediated apoptosis. (E) Macrophages were treated as in (D), except apoptosis was determined 16 h after TRAIL exposure by ELISA for apoptotic histone-associated cellular DNA fragmentation in macrophage lysates (cell death ELISA; error bar, SD).

**Figure 6:**
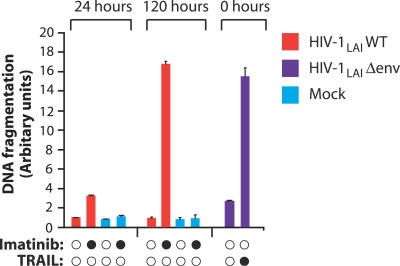
Prolonged Exposure to Imatinib Alone Is Sufficient to Induce Apoptosis in HIV-1–Infected Macrophages Five days after infection with HIV-1_LAI_ wild-type or Δenv viruses, macrophages were incubated with Imatinib for 24 or 120 h, and levels of apoptosis were determined by ELISA for histone-associated cellular DNA fragmentation in macrophage lysates (cell death ELISA; error bars, SD).

The aforementioned results predict that agents that interfere with the function of M-CSF or of its receptor would antagonize HIV-1′s ability to protect infected macrophages from TRAIL-mediated killing. The anti-cancer drug Imatinib inhibits the tyrosine kinase activity of the bcr-abl oncogene and also shows considerable selectivity and efficacy toward the intrinsic tyrosine kinase activity of the M-CSF receptor [27]. Therefore, we examined Imatinib for its potential to kill HIV-1–infected macrophages. The M-CSF receptor undergoes autophosphorylation after ligand binding [27]. At concentrations obtainable in vivo, Imatinib inhibited receptor phosphorylation in primary human macrophages (Figure 5A), indicating that Imatinib blocked signaling by the M-CSF receptor in human macrophages. Furthermore, while TRAIL-R1 expression in wild-type virus–infected macrophages was similar to uninfected macrophages, addition of Imatinib increased TRAIL-R1 expression to levels similar to that of Δenv-infected macrophages (Figure 5B and 5C). The elevation of TRAIL-R1 expression on wild-type–infected macrophages by Imatinib was consistently observed on cells from three donors (*p* < 0.001), and Imatinib did not alter TRAIL-R1 levels on macrophages infected with an envelope-minus variant (Figure 5C). Furthermore, Imatinib had no effect on TRAIL-R1 expression in uninfected macrophages (Figure 5B and 5C). Imatinib restored the sensitivity of wild-type HIV-1–infected macrophages to TRAIL and augmented TRAIL susceptibility beyond that of Δenv-infected macrophages. Using Annexin V and propidium iodide to stain for apoptotic cells, the addition of Imatinib followed by TRAIL caused HIV-1–infected macrophages to undergo apoptosis (Figure 5D). In the absence of stimuli, infected macrophages underwent a low level of apoptosis, which was not enhanced by TRAIL. Importantly, Imatinib, TRAIL, or their combination did not promote TRAIL-R1 expression or apoptosis in uninfected macrophages present in the cultures (Figure 5D). Analysis of apoptosis in HIV-1–infected macrophages by DNA fragmentation further confirmed that Imatinib negated the resistance of wild-type–infected macrophages to TRAIL and facilitated extensive apoptosis, equivalent to macrophages infected with a Δenv virus and exposed to TRAIL (Figure 5E). Macrophages infected with an R5-tropic wild-type HIV-1 were similarly resistant to TRAIL-mediated apoptosis. Imatinib likewise rendered these cells highly sensitive to apoptosis by TRAIL (Figure S4D). Imatinib alone was sufficient to counteract the survival signals that keep HIV-1–infected macrophages alive, and long-term exposure to Imatinib resulted in apoptosis of wild-type–infected macrophages even in the absence of TRAIL (Figure 6). This finding was reflected by a decrease in Mcl-1 expression and a further increase in pro-apoptotic protein expression (unpublished data). Overall, these data suggest that Imatinib may be an effective strategy to induce the TRAIL-dependent apoptotic death of HIV-1–infected macrophages in vivo. Our results also raise the intriguing possibility that Imatinib may be able to promote the death of infected macrophages without the need for TRAIL.

## Discussion

**Figure 7:**
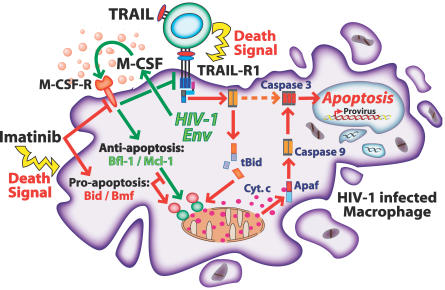
Experimental Model: Following HIV-1 Infection*,* De Novo Synthesized Viral Envelope Induces the Release of M-CSF M-CSF opposes TRAIL by suppressing TRAIL-R1 expression and upregulating the anti-apoptotic factors Bfl-1 and Mcl-1. The anti-cancer drug Imatinib disables the protective effect of envelope by inhibiting M-CSF activity. Upon inhibition of M-CSF activity, TRAIL-R1 expression is increased and the action of Bfl-1 and Mcl-1 is blocked by pro-apoptotic bcl-2 family proteins. Further exposure of infected macrophages to Imatinib for several days induces apoptosis in the absence of TRAIL and is associated with increased pro-apoptotic protein expression.

In this study, we present evidence for a novel envelope-dependent mechanism that allows HIV-1–infected macrophages to persist in the face of apoptotic clearance processes. Furthermore, we describe a chemotherapeutic agent, Imatinib, which has the potential to disable this viral defense. Figure 7 summarizes the mechanisms employed by the HIV-1 envelope glycoprotein to subvert the host apoptotic response to TRAIL in infected macrophages, as well as those processes countered by Imatinib to restore TRAIL sensitivity and induce apoptosis. Apoptosis by TRAIL can be propagated by signals via intrinsic or extrinsic pathways [24]. However, TRAIL-mediated apoptosis in macrophages was dependent upon the extrinsic or mitochondrial pathway of apoptosis. Despite the HIV-1 envelope-dependent upregulation of IAP family proteins (which can block the extrinsic pathway) early in infection, signals from the TRAIL receptor that promoted apoptosis through the mitochondrial pathway were dominant in HIV-infected macrophages. This finding agrees with Zheng et al. [28], who report that human primary monocytes resist TRAIL-mediated apoptosis through Bcl-2–dependent (mitochondrial) mechanisms. The exact molecular basis for the regulation of pro-survival and anti-apoptotic factors by HIV-1 envelope is under investigation through mutagenesis studies and analysis of signal transduction pathways influenced by envelope expression.

The dissemination of HIV-1 from infected macrophages to neighboring T lymphocytes requires intimate cell–cell contact, which can leave infected macrophages susceptible to apoptosis by death ligands on lymphocytes. TRAIL cytotoxicity is mediated effectively by CD4^+^ T lymphocytes and by CD8^+^ T cells and NK cells [29,30]. In vivo, macrophages can undergo apoptosis after activating CD4^+^ T cells. The induction of macrophage apoptosis by TRAIL on CD4^+^ T cells has been proposed to form part of macrophage homeostasis during antigen presentation [4]. The regulation of activated macrophages by TRAIL also serves to limit immune responses and to target macrophages infected with intracellular organisms for elimination [31]. For these reasons, the avoidance of apoptosis by TRAIL is likely an important process for the survival of HIV-1–infected macrophages in vivo. Macrophages infected by HIV-1 did not express receptors for other death ligands; neutralizing TRAIL—but not the Fas ligand or TWEAK—on T lymphocytes prevented the apoptosis of infected macrophages when the protective functions of the viral envelope were disabled. These findings suggest TRAIL is a major restriction for the persistence of HIV-1 in macrophages during interactions with immune cells.

Mechanisms that restrict TRAIL-mediated apoptosis have been described for several unrelated viruses. Gamma-herpesviruses, including Kaposi sarcoma-associated human herpesvirus-8, encode FLICE-inhibitory proteins (FLIPs) that interact with the adaptor protein FADD to inhibit the generation of active caspase 8, which is necessary to trigger apoptosis by TRAIL [32]. Human adenovirus type-5 encodes three proteins (RID) that induce the internalization from the cell surface and lysosomal degradation of TRAIL receptors [10]. Human T cell leukemia virus type 1–infected T cell lines are also resistant to TRAIL-mediated apoptosis, presumably because of activation of TRAIL expression by the viral transactivator Tax [33]. Apoptosis of B cells by TRAIL is inhibited by the Epstein-Barr virus–encoded BHRF1 protein, which functions in the apoptotic pathway similarly to the host protein Bfl-1 that is induced by M-CSF in HIV-1–infected macrophages [34,35]. TRAIL-R1 is specifically downregulated in cells infected by human herpesvirus 7 and is associated with their resistance to TRAIL-mediated cytotoxicity [36]. Our demonstration that HIV-1 envelope glycoprotein also counteracts TRAIL-mediated apoptosis underscores the general importance of evading the immune pressure exerted by TRAIL in order for viruses to persist in the infected cell.

## Materials and Methods

### Antibodies and reagents.

Neutralizing antibody to human M-CSF and isotype control antibody were used at 10 μg/ml^−1^ and obtained from R & D Systems. ELISA kits for human M-CSF, recombinant soluble TRAIL-R1, and control receptor proteins were also supplied by R & D Systems. HSA (murine CD24), Fas and TWEAK-R antibodies for flow cytometry, and antibodies to CD3 and CD28 were purchased from BD Pharmingen. TRAIL-R1/DR4 antibody and soluble human recombinant TRAIL were obtained from Axxora LLC. Monoclonal and polyclonal antibodies to the M-CSF receptor and antibodies to phosphotyrosine and tubulin were purchased from Santa Cruz Biotech. Antibodies to Bfl-1 and Mcl-1 were supplied by Cell Signaling Technologies. Imatinib mesylate was obtained from Sequoia Research Products.

### Cells and viruses.

The R5 tropic virus HIV-1_ADA_ was prepared as detailed previously [18,37]. For the preparation of HIV-1 X4-tropic LAI and HSA viruses pseudotyped with the VSV envelope, 293 T cells were co-transfected with 25 μg of HIV-1 DNA and 25 μg of a VSV-G expression vector by Calcium Phosphate precipitation. Viruses containing supernatants were harvested 60 h post-transfection and standardized by RT assay as described previously [18,37]. HIV-1_HSA_ [38] contains the mouse gene CD24, a heat stable surface antigen, in place of Vpr. As a result, HIV-1_HSA_–infected cells can be identified by flow cytometry, and pseudotyping with VSV-G envelope effectively bypasses the requirement for Vpr in macrophage infection. Envelope-minus HIV-1_LAI_ and HIV-1_HSA_ variants were constructed by Nde I restriction site fill-in. HIV-1_LAI_ΔCD4b contains a two amino acid deletion in a critical CD4-receptor binding domain of HIV-1 envelope that disrupts CD4 binding [21]. Lymphocytes and monocytes were obtained by leukapheresis from normal donors seronegative for HIV-1 and hepatitis B. Populations of CD8^+^ T lymphocytes were obtained by additional purification with antibody-coated magnetic beads according to manufacturer's instructions (Dynal-Invitrogen). The lymphocytes were activated with antibodies to CD3 and CD28 (5 μg/ml^−1^ each) for 48 h and washed in medium prior to co-culture experiments with macrophages. Monocytes were further separated by countercurrent centrifugal elutriation as detailed elsewhere [39]. Elutriated monocytes were differentiated from macrophages by culture for 4 d in medium containing M-CSF (R & D Systems) and for a further 3 d in medium without M-CSF. Macrophages were then used for virus infections within 1–5 d.

### Apoptosis, cell death, and viability assays.

ELISAs for determining apoptosis by the cleavage of caspase 3 (to active form) were obtained from Cell Signaling Technologies. Briefly, 1.4 × 10^6^ macrophages infected with wild-type and envelope-minus viruses were lysed according to the supplier's protocol 1 h after the addition of soluble recombinant TRAIL (100 ng/ml^−1^). Protein content was determined by Bradford Assay (Bio-Rad), and 220 μg of protein in 200 μl from each culture was assayed in ELISA. Reagents were purchased from BioVision and used according to the supplier's protocol for the measurement of apoptosis by Annexin V and propidium iodide staining in flow cytometry. Apoptotic DNA fragmentation was measured by ELISA for histone-associated DNA fragments present in macrophage lysates prepared according to the manufacturer's instructions (Roche) 16 h after exposure to apoptotic stimuli. For the determination of macrophage longevity, cell death was quantitated by ELISA for the release of histone-associated fragmented DNA into macrophage culture supernatants (Roche). Briefly, after the addition of soluble TRAIL to 0.7 × 10^6^ cells, 10 μl of supernatant was harvested over time for ELISA. Supernatant from macrophages treated for 72 h with 100 μM Apoptosis Activator I (EMD Biosciences), which resulted in comprehensive visual cell death, was used as a positive control and as a value for remaining viable cells calculated by subtraction. In other experiments, an MTT assay (Sigma) was used to measure cell viability.

### T cell–mediated inhibition of viral replication.

Infected macrophages were monitored by RT assay until viral replication approached peak. The medium was changed, and anti-CD3/CD28 stimulated autologous CD8^+^ T cells (2.5 × 10^6^) after incubation with soluble TRAIL-R1 or control receptor for 1 h were co-cultured with infected macrophages for 4 h. The CD8 cells were then gently removed and virus production from macrophages determined by RT assay 24 h later.

### RNA interference and quantitation.

Macrophages, 1.4 × 10^6^, were infected with wild-type HIV-1 LAI and a Δenv variant and monitored by RT assay until viral replication approached peak levels. Macrophages were transfected with Lipofectamine 2000 containing 100 nM duplexed small interfering RNA (siRNA) to Bfl-1, Mcl-1, or scrambled siRNA (Dharmacon) for 4 h, then re-fed conditioned medium from uninfected macrophages. The transfection was repeated the next day. Cells were harvested for ELISA or RNA analysis 16 h after the second transfection. RNA interference (RNAi)–mediated mRNA decay was assessed on total RNA from 200,000 cells prepared by Trizol (Invitrogen) in SyBr Green real time RT-PCR (Quantitect SyBr Green Kit; Qiagen) using gene-specific primers from SuperArray. The levels of cellular mRNAs were similarly determined by SyBr Green real-time PCR or by ribonuclease protection assay (BD Pharmingen), as described previously [37].

### Statistical analyses.

The statistical significance of data, where indicated, was determined by ANOVA or *t*-test using Prism 5 (GraphPad Software). Mean values ± SEM are shown graphically; annotations indicate the confidence level (*p*-value) and number of replicates (*n*).

## Supporting Information

Figure S1 M-CSF Production by Infected Macrophages Is Not Dependent on Envelope-CD4 Interaction Virus production (A) and M-CSF release (B) were examined following infection with pseudotyped HIV-1 variants containing intact or deleted envelope genes or an HIV-1 mutant (HIV-1_LAI_ΔCD4b) lacking a functional CD4 receptor binding motif in envelope. Cumulative M-CSF release (C) during the course of viral replication was determined by normalizing the amount of M-CSF to RT output (error bars, SD). (D) The incubation of cell-free HIV-1 virions with macrophages does not promote M-CSF release. R5-tropic HIV-1_ADA_WT and HIV-1_ADA_Δenv viruses were VSV-G pseudotyped and were purified on continuous 15%–60% sucrose gradients. Individual gradient fractions were dialyzed, analyzed for RT activity, and added to macrophage cultures for 1 h. M-CSF production was determined after 16 h by ELISA. Gradient fractions of mock-infected macrophage supernatants were used as controls.
(356 KB PDF)

Figure S2 HIV-1 Envelope Regulates Host Genes Involved in Apoptotic Pathways Messenger RNA levels were compared in cDNA gene arrays between macrophages infected with pseudotyped X4 wild-type HIV-1 and an envelope-minus variant 5 d post-infection. Gene expression was considered significantly different when the variation was ≥1.7 units [40].
(259 KB PDF)

Figure S3 Macrophages Exhibit Moderate Resistance to TRAIL at Intervals Post-Infection when M-CSF Levels in Culture Supernatants Are Low (A) M-CSF levels in HIV-1 wild-type– and Δenv-infected macrophages at different intervals post-infection. M-CSF induction is not apparent during the first 4 d post-infection.
(B) Sensitivity of infected macrophages to TRAIL at 2 d (no M-CSF in culture supernatants) and 8 d (elevated M-CSF in culture supernatants) post-infection (error bars, SD).
(C) Analysis of apoptosis-related gene expression at different intervals post-infection. Three anti-apoptotic genes (cIAP-1, cIAP-2, XIAP) were upregulated in an HIV-1 envelope-dependent manner even at 2 d post-infection when M-CSF levels in culture supernatants were undetectable. mRNA levels were determined by quantitative RT-PCR.
(352 KB PDF)

Figure S4 Macrophages Infected with R5-tropic HIV-1_ADA_ Regulate TRAIL-R1 Expression Via M-CSF Release and Succumb to TRAIL-Mediated Apoptosis Only in the Presence of Imatinib (A) HIV-1_ADA_ induced infected macrophages to release M-CSF during viral replication.
(B) TRAIL-R1 expression on HIV-1_ADA_ wild-type– or mock-infected macrophages was analyzed by flow cytometry 16 h after treatment with recombinant M-CSF (5,000 pg/ml^−1^) or with a neutralizing antibody to M-CSF (error bars, SD).
(C) Imatinib renders HIV-1_ADA_ wild-type–infected macrophages sensitive to TRAIL-mediated apoptosis. HIV-1_ADA_ wild-type– and mock-infected macrophages were incubated with Imatinib for 16 h and stimulated with TRAIL. Apoptosis was determined by ELISA for active (cleaved) caspase 3 (error bars, SD).
(308 KB PDF)

## Abbreviations

M-CSF: macrophage colony-stimulating factor
RNAi: RNA interference
RT: reverse transcriptase
siRNA: small interfering RNA
SHIV: SIV/HIV chimera
SIV: simian immunodeficiency virus
TRAIL: tumor necrosis factor–related apoptosis-inducing ligand
TWEAK: tumor necrosis factor–like weak inducer of apoptosis
VSV: vesicular stomatitis virus
