# Supplementary figures and images for: Apoptotic Killing of HIV-1–Infected Macrophages Is Subverted by the Viral Envelope Glycoprotein
Source: PLoS Pathog. 2007 Sep 28;3(9):e134. doi: 10.1371/journal.ppat.0030134 (PMC2323301; doi:10.1371/journal.ppat.0030134)

Figure S1

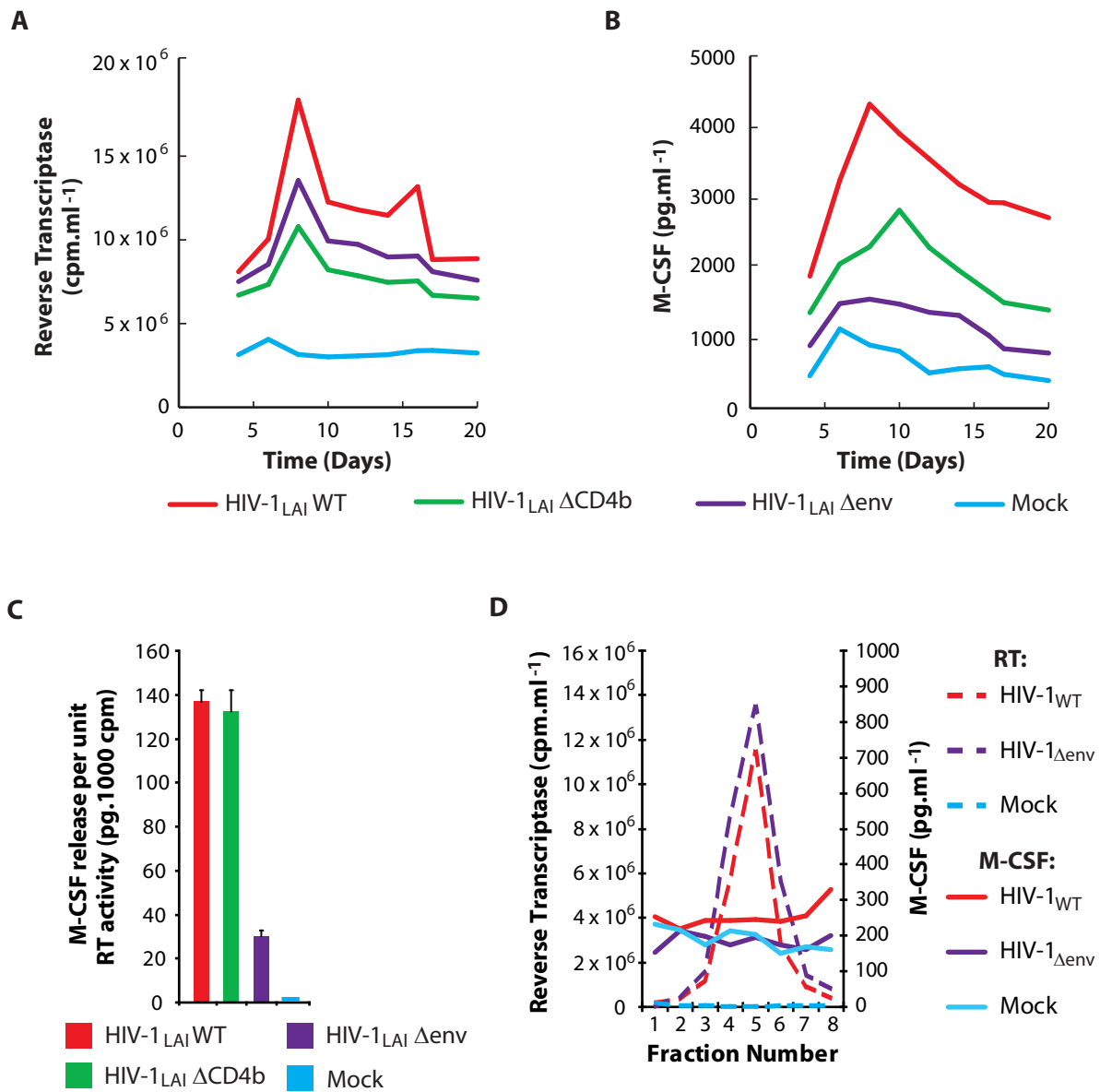

Supplement: Figure S1 — Virus production (A) and M-CSF release (B) were examined following infection with pseudotyped HIV-1 variants containing intact or deleted envelope genes or an HIV-1 mutant (HIV-1LAIΔCD4b) lacking a functional CD4 receptor binding motif in envelope. Cumulative M-CSF release (C) during the course of viral replication was determined by normalizing the amount of M-CSF to RT output (error bars, SD). (D) The incubation of cell-free HIV-1 virions with macrophages does not promote M-CSF release. R5-tropic HIV-1ADAWT and HIV-1ADAΔenv viruses were VSV-G pseudotyped and were purified on continuous 15%–60% sucrose gradients. Individual gradient fractions were dialyzed, analyzed for RT activity, and added to macrophage cultures for 1 h. M-CSF production was determined after 16 h by ELISA. Gradient fractions of mock-infected macrophage supernatants were used as controls. (356 KB PDF) [file ppat.0030134.sg001.pdf]

Figure S2

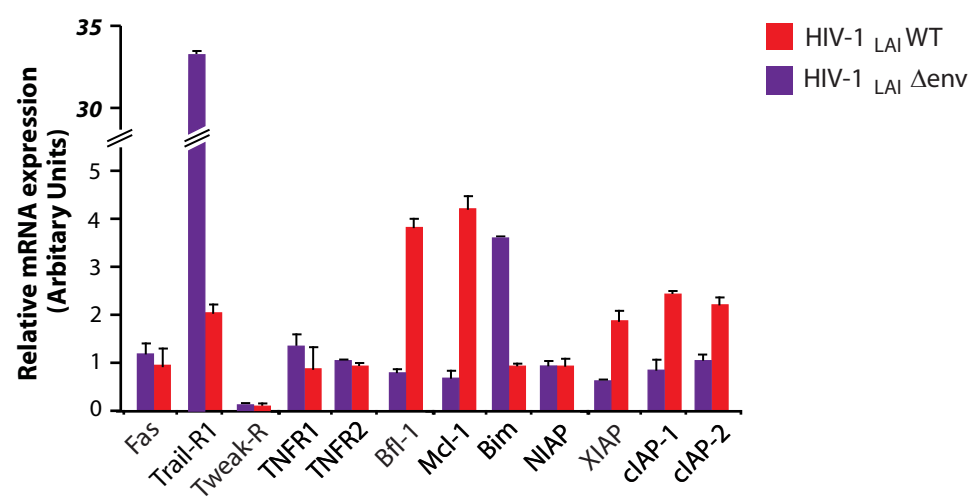

Supplement: Figure S2 — Messenger RNA levels were compared in cDNA gene arrays between macrophages infected with pseudotyped X4 wild-type HIV-1 and an envelope-minus variant 5 d post-infection. Gene expression was considered significantly different when the variation was ≥1.7 units [40]. (259 KB PDF) [file ppat.0030134.sg002.pdf]

Figure S3

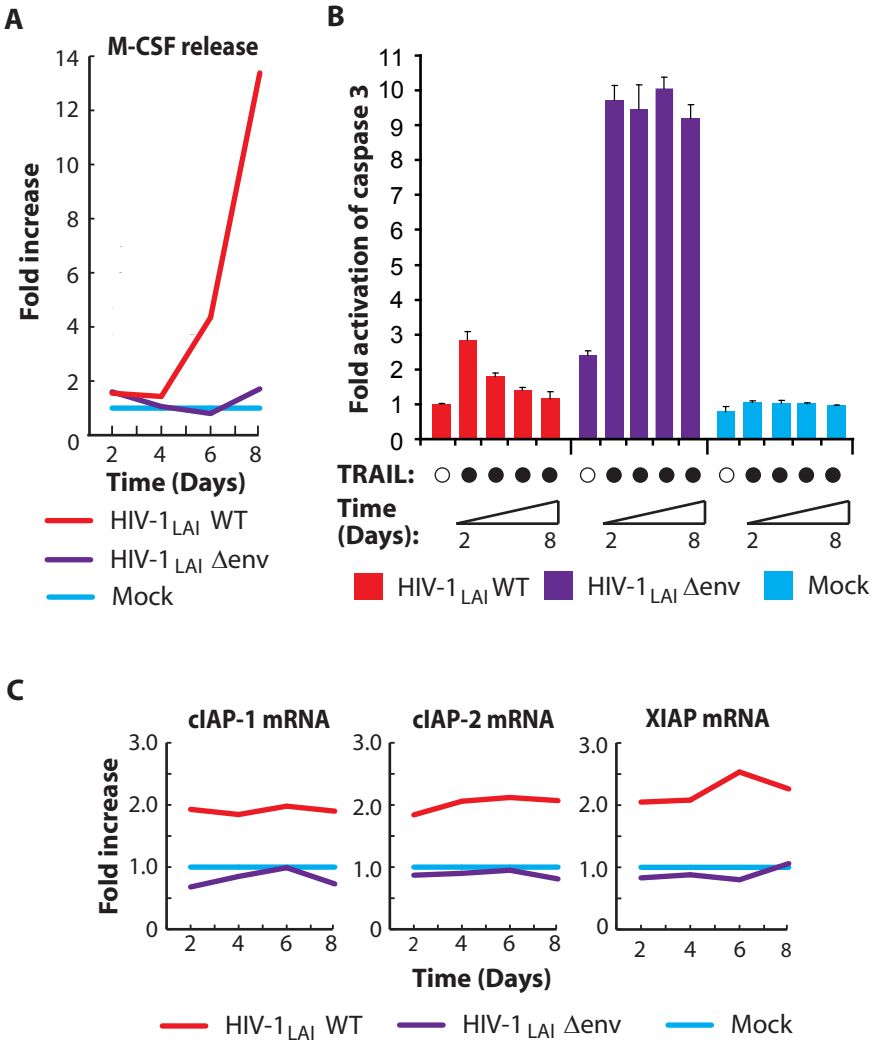

Supplement: Figure S3 — (A) M-CSF levels in HIV-1 wild-type– and Δenv-infected macrophages at different intervals post-infection. M-CSF induction is not apparent during the first 4 d post-infection. (B) Sensitivity of infected macrophages to TRAIL at 2 d (no M-CSF in culture supernatants) and 8 d (elevated M-CSF in culture supernatants) post-infection (error bars, SD). (C) Analysis of apoptosis-related gene expression at different intervals post-infection. Three anti-apoptotic genes (cIAP-1, cIAP-2, XIAP) were upregulated in an HIV-1 envelope-dependent manner even at 2 d post-infection when M-CSF levels in culture supernatants were undetectable. mRNA levels were determined by quantitative RT-PCR. (352 KB PDF) [file ppat.0030134.sg003.pdf]

Figure S4

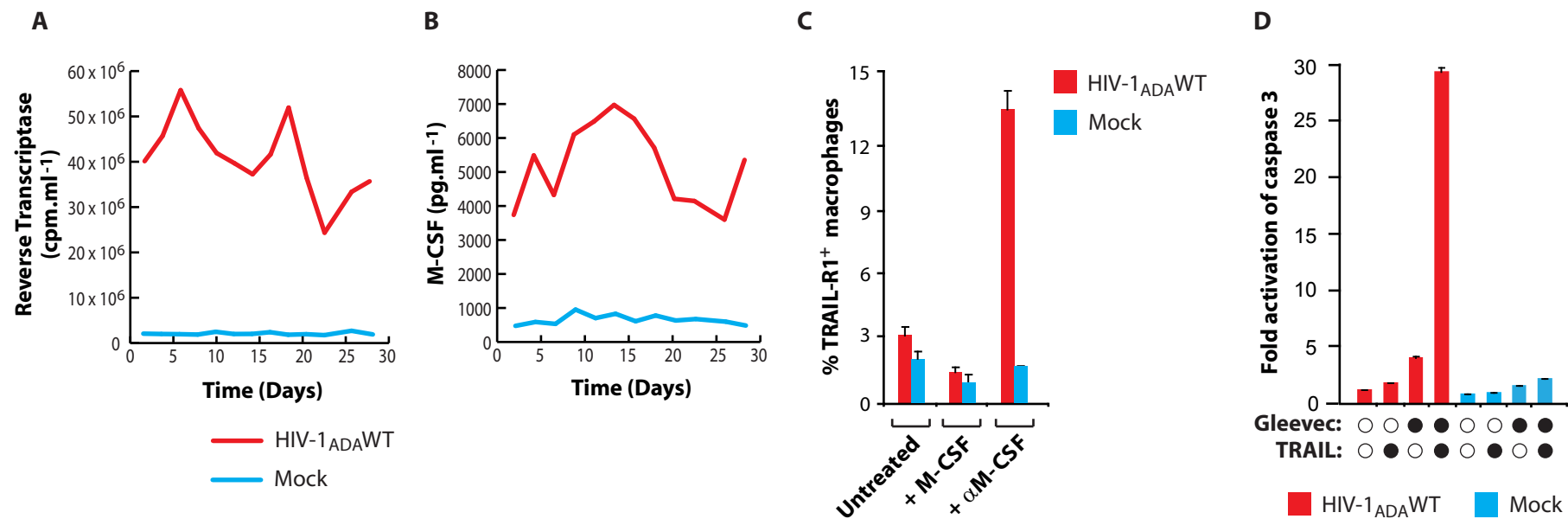

Supplement: Figure S4 — (A) HIV-1ADA induced infected macrophages to release M-CSF during viral replication. (B) TRAIL-R1 expression on HIV-1ADA wild-type– or mock-infected macrophages was analyzed by flow cytometry 16 h after treatment with recombinant M-CSF (5,000 pg/ml−1) or with a neutralizing antibody to M-CSF (error bars, SD). (C) Imatinib renders HIV-1ADA wild-type–infected macrophages sensitive to TRAIL-mediated apoptosis. HIV-1ADA wild-type– and mock-infected macrophages were incubated with Imatinib for 16 h and stimulated with TRAIL. Apoptosis was determined by ELISA for active (cleaved) caspase 3 (error bars, SD). (308 KB PDF) [file ppat.0030134.sg004.pdf]
